# Acceptability and Effectiveness of the Storekeeper-Based TB Referral System for TB Suspects in Sub-Districts of Lilongwe in Malawi

**DOI:** 10.1371/journal.pone.0039746

**Published:** 2012-09-04

**Authors:** Bertha Nhlema Simwaka, Sally Theobald, Annie Willets, Felix M. L. Salaniponi, Patnice Nkhonjera, George Bello, Stephen Bertel Squire

**Affiliations:** 1 Research for Equity and Community Health Trust, Lilongwe, Malawi; 2 Liverpool School of Tropical Medicine, Pembroke Place, Liverpool, United Kingdom; 3 Wellsense International Public Health Consultants, Kilifi, Kenya; 4 Malawi National TB Control Programme, Lilongwe, Malawi; 5 Research for Equity and Community Health Trust, Lilongwe, Malawi; Tulane University School of Public Health and Tropical Medicine, United States of America

## Abstract

**Background:**

Early access to tuberculosis diagnosis and treatment remains a challenge in developing countries. General use of informal providers such as storekeepers is common. The aim of this study was to determine the effectiveness and acceptability of a storekeeper-based referral system for TB suspects in urban settings of Lilongwe, Malawi.

**Methods:**

The referral system intervention was implemented in two sub-districts. This was evaluated using a pre and post comparison as well as comparison with a third sub-district designated as the control. The intervention included training of storekeepers to detect and refer clients with chronic cough using predesigned referral letters along with monitoring and supervision. Data from a community based chronic cough survey and an audit of health centre records were used to measure its effectiveness. Focus group discussions and in-depth interviews were carried out to document acceptability of the intervention with the different stakeholders.

**Results:**

Following the intervention, the mean patient delay appeared lower in the intervention than comparison areas (2.14 weeks (SD 5.8) vs 8.8 weeks (SD 15.1)). However, after adjusting for confounding variables this difference was not significant (p = 0.07). After the intervention the proportion of the population diagnosed with smear positive TB in the intervention sites (1.2 per 1000) was significantly higher than in the comparison area (0.6 per 1000, p<0.01) even after adjusting for sex and age. Qualitative findings suggested that (a) the referral letters triggered health workers to ask patients to submit sputum for TB diagnosis (b) the approach may be sustainable as the referral role was linked to the livelihood of the storekeepers.

**Conclusion:**

The study suggests that the referral system with storekeepers is sustainable and effective in increasing smear positive TB case notification. Studies that assess this approach for control of other diseases along with collection of specimens by storekeepers or similar providers are needed.

## Introduction

Despite progress made in achieving treatment success rates in most countries, tuberculosis remains a major global health burden. Many people suffering from TB are still not accessing diagnosis and treatment due to health system and socio-economic barriers [1]–[2]. These barriers interplay to threaten attainment of the UN Millennium Development Goals aimed at halting and beginning to reverse the incidence of TB and reducing by 50 percent the prevalence and mortality of TB by 2015. Globally tuberculosis (TB) case detection is still lower than the 70 percent target [1]. As in most resource-poor settings, the case detection rate in Malawi is about 50 percent [1].

Poverty related issues influence or hinder early access to diagnosis and treatment at the household level. This leads to community members seeking care from providers outside of the formal TB control system. For example, patients use informal providers such as storekeepers who are convenient from a financial and opportunity cost perspective [3]. There are also system related factors such as delay in early referral or transfer of patients between departments for TB diagnosis by health workers at facility levels, leading to repeated visits to health facilities before diagnosis [4]–[5]. The interplay between poverty and health system factors leads to long care-seeking pathways, and high direct and indirect costs among poor men and women. For example in urban Malawi, it has been documented that the cost among poor people of accessing TB services can be as high as 248% of their monthly income [6].

In many countries there are formal and informal providers outside of the publically funded routine health service that can be engaged to accelerate achievement of global and national targets [7]. Innovations both to reach poor communities and accelerate decisions by health workers are urgently required. Community-based health interventions are widely believed to contribute to promoting equity in access to health services and social justice [8].

Investigation of case detection and referral of TB suspects by providers beyond the public health system has been documented for formal private health providers [9]–[10]. However, in countries like Malawi, formal private health providers are largely found in urban and peri-urban areas and are out of reach - both financially and geographically - of poorer and vulnerable groups. In contrast, storekeepers are found in all rural and urban communities in Malawi. The storekeepers have an established role in selling groceries and simple medicines, including remedies for cough. They have a recognized place within the broader social system under village or community leaders. To date no formal partnership for the early diagnosis has been explored between storekeepers and the public health sector. There are examples of successful partnerships between storekeepers and malaria control programmes which have increased access to treatment of uncomplicated malaria in Kenya and Tanzania [11]–[12].

Expanding the role of storekeepers to include identification and referral of TB suspects could increase opportunities for early diagnosis and treatment for poor men and women. In this paper we present findings about the effectiveness and acceptability of a health system intervention which was implemented with storekeepers between 2003 and 2006. The objectives were; (i) to explore the feasibility and effectiveness of establishing a referral system with store keepers and (ii) to improve their advisory skills on tuberculosis. The outcome measures were reduction in patient delay for people suffering from chronic cough, proportion of TB diagnoses at the health facility which were referred by storekeepers, number of chronic cough and smear positive cases notified per 1000 head of population, and proportion of chronic cough cases referred by storekeepers. Patient delay was defined as time between onset of cough and visit to a TB diagnostic facility. Societal and providers' perceptions of the role of storekeepers before and after the intervention, factors that led to acceptability of the role of storekeepers and challenges faced in implementing the intervention were also documented.

## Materials and Methods

Data for the outcome measures were collected before and after introduction of the intervention in two sub-districts of Lilongwe; Ngwenya and Kauma. The same data were collected at the same time points in a control sub-district, Chinsapo, where no intervention was implemented. These locations were selected on the basis of similarities in poverty level, low utilization of TB services and poor sanitation as outlined in Table 1. The work was carried out by a consortium consisting of the National TB Control Programme (NTP), Liverpool School of Tropical Medicine through the EQUI-TB Knowledge Programme, the Lilongwe District Health Office (DHO), the City Assembly, community health committees and leaders.

**Table 1:** Poverty Characteristics of the three studied areas.

Area	Population size	Secondary school education	Female headed households	Proportion employed in the formal sector	Poverty head-count	Proportion of households using electricity for light
Ngwenya	13602	7%	25%	30–60%	50%	<4.8%
Kauma	13203	7%	25%	30–60%	64%	4.8–10%
Chinsapo	34692	7%	25%	30–60%	50%	<4.8%

Source: Projections based on Malawi 1998 Census and indicators are from Malawi – An Atlas of Social Statistics (2002).

### Description of the intervention

The intervention was developed and implemented in a participatory manner. The key components of the process and package were:

#### Developing a participatory approach and structures

The Lilongwe City Assembly health department facilitated establishment of structures that were used to develop the intervention package. The structures were created to ensure participation of all stakeholders including community health committees, storekeepers and community leaders [13]. The process of developing the intervention required consensus building [13]–[14] and took almost 15 months to complete. The structures established were:

#### The project coordinating and design group

The members were the Information, Communication, and Education Officers from the National TB Control Programme, the Lilongwe District TB Officer, Lilongwe City Assembly TB Officer, two community leaders and two members of community health committees. This group was responsible for developing the intervention package and training storekeepers, orientation of community leaders, health committees and volunteers about the role of storekeepers.

#### Community-based monitoring group

The members were health surveillance assistants responsible for the communities, two representatives of trained storekeepers, a representative of the market committee, community health committee members and community leaders. Their role was to monitor the performance of trained storekeepers, review progress and address challenges. This group reported to the project coordinating group during the review meetings.

#### The intervention package for storekeepers

The intervention package included job aides for storekeepers, referral letters, training manuals and flip charts of pictures and information for training. The content of each component is outlined in Table 2 below. The content was informed by: (a) National TB Control policy, manuals and guidelines, (b) evidence on different community members' perceptions [15], (c) feedback from policy makers, researchers and community members and (d) a pilot of the intervention materials in a sub-district where the research did not take place. The content of training manuals included general knowledge on tuberculosis, misconceptions about the disease, skills-building on communication, identifying and referring the TB suspects using cough of more than 3 weeks and business management.

**Table 2:** Intervention Package.

Intervention Package Component	Content
A. Trainers Guide	
A.1 General	1. Definition of participatory teaching methods: role plays, group work, brainstorming. 2. Supervision plan and tools after training. 3. Monitoring plan and tools after training
A.2 Storekeeper training day 1	1. Cause and symptoms of TB. 2. Misconceptions about TB. 3. Diagnosis and treatment of TB. 4. Investigation and diagnosis of TB. 5. Discussion on their perceived role. 6. Outline of expected role in referring their clients
A.3 Storekeeper training day 2	1. Communication skills with clients, storekeepers, community health Committee. 2. How to identify and refer clients suspected to be suffering from TB. 3. Discussion on their worries, fears and how to handle challenges. 4. Business management, sales/business record keeping. 5. Record keeping for chronic cough referrals by storekeepers
A.4 Storekeeper training day 3Practice and review	1. Practicing on probing and advisory skills on chronic cough. 2. Practice how to ask questions to clients. 3. Review, feedback from participants and facilitators. 4. Evaluation of training
B. Flip Chart	Pictures illustrating TB messages about transmission, symptoms and care seeking: 1. Cough for more than 3 weeks could be TB disease. 2. Investigation of chronic cough should be sought at hospital or health centre with smear microscopy. 3. And that TB can be cured with drugs available free of charge at public health facilities
C. Referral System	
C1. Storekeepers	1. Standard referral letter 2. Record Book for referrals made 3. Small flipchart books 4. Algorithms for referral decision
C2. Health Facility	1. File for referrals received from clients from storekeepers 2. Orientation of health workers on referrals by storekeepers
C3. Review meetings	Quarterly review meetings with representatives from health facility, storekeepers and community leaders

### Recruitment of storekeepers

The eligibility criteria which were agreed upon by health workers and community leaders for recruiting storekeepers were:

- The storekeepers were known and registered with the market chairpersons and the community leaders.
- They had operated within the community for more than 12 months
- The storekeepers accepted voluntarily to participate in the intervention with no financial incentives.

There were 110 storekeepers in the two intervention areas, 64 met the eligibility criteria and were included in the training. There were 102 storekeepers in the control area and none were trained. The consent was also obtained, where necessary, from the shop owners. Most shops were owned and managed by men; only seven women storekeepers were trained.

### Training of store keepers

Members of the project coordinating and design group were also responsible for the training of storekeepers for three days. Training was undertaken using the training manual and flip charts. The training was done using the participatory techniques as outlined in the training manuals (Table 2) to build referral and advisory skills. Techniques used were interactive role plays, group work, brainstorming and a field practical in health promotion and referring of patients. Applications of these techniques were described in the training manuals.

Three days were used for training as outlined in Table 2. The first two days were classroom based combined with role plays. On the final day, trainees practiced their new skills with clients under supervision in a number of stores.

In addition issues of business management, record keeping and marketing of products were covered by a Social Welfare Officer from the City Assembly. This was conducted for shop keepers to improve sales, record keeping and product marketing as an incentive for their participation.

Evaluation of the training was done by getting feedback from participants in group discussions. Follow-up supervisions of storekeepers were carried out by the trainers and health surveillance assistants to support the providers and identify areas to be covered in refresher courses. The refresher courses addressed gaps identified by trainers during supervision and this focused on areas which were related to the core roles of the providers such as referral and health promotion skills.

### Implementation of the intervention by storekeepers

The storekeepers were responsible for screening customers seeking medicines and provided the referral letters outlined in table 2. The index was any person with a cough seeking to buy medicine; storekeepers probed for duration and provided advice to those with a cough of more than three weeks on the need to visit a health centre for appropriate diagnosis in line with national policy. The follow-up supervisions of storekeepers, volunteers and community health committees were carried out by the trainers and health surveillance assistants and findings were incorporated into the yearly 1 day refresher trainings.

## Measuring Effectiveness and Documenting Acceptability

A multi-method approach was used to measure effectiveness which included quantitative methods using both routine and survey data and qualitative methods to explore different stakeholder's perception and experience of the intervention.

### Quantitative methods

The two approaches used to measure effectiveness:

1. A pre & post household survey,
2. And an audit of chronic cough and treatment registers

#### Chronic cough household surveys

The community based chronic cough surveys were conducted in both intervention and comparison sites pre and post-intervention to collect information on pathways to care seeking. Each survey round was designed to obtain a minimum sample of 107 per category (intervention or comparison). Previous studies have shown that TB patient delay is approximately 8 weeks from symptom onset to TB diagnosis [16]. With 107 patients with chronic cough identified in each category, the study had the power to detect a reduction in mean patient delay of 50% from 8 to 4 weeks at 95% confidence level and power of 90%.

The intervention and comparison sites are sub-divided under elected community leaders, and these sub-divisions were used as clusters for the survey. All households in each cluster were listed and the first household was randomly selected by the research supervisor and systematic sampling of every second household was used for selection of subsequent households. In the selected households a head or adult member was interviewed to identify members suffering from a cough of more than three weeks, who were then interviewed using a pre-tested structured questionnaire. Data collection was carried out by a team of trained research assistants during the months of November 2003 to May 2004 and again from November 2005 to May 2006.

#### Audit of Routine health Centre Based Data

The audit of routine health center based data on chronic cough and tuberculosis was also undertaken to assess the impact of the intervention on the TB suspects and cases detected from the study and comparison areas. We audited the entire routine chronic cough and treatment registers in the whole of urban Lilongwe District for place of residence in order to quantify TB suspects and smear positive TB cases notified from the intervention and comparison areas. All health facilities were audited because patients can access any health facility regardless of geographical reach within urban Lilongwe. The pre-intervention audit was performed on data recorded in the registers between 1^st^ January and 31^st^ December 2003 and the post-intervention audit was on data recorded between 1^st^ January and 31st December 2006.

### Statistical analysis

Double data entry and cleaning of survey and register data was carried out using Epi-info version 6.04d. STATA version 10 was used for data analysis. To adjust for covariates, generalised linear modelling with log link and family Poisson was used to measure the impact of the intervention on patient delay. The poverty level of the survey participants was assessed by the proxy measure developed using data from the 1998 integrated household survey for urban Lilongwe [17].

### Qualitative Component

The qualitative evaluation was based on naturalistic inquiry suitable for describing and analysing community based interventions within a social setting [18]. This was used to document factors that underpinned any observation in the practices of storekeepers and experiences of access to TB diagnosis by community members. Focus group discussions and in depth interviews were carried out simultaneously with implementation of the intervention, documenting views at baseline, during and post-intervention from 2003 to 2007. Non-probability sampling was used to recruit the participants, who had an in-depth knowledge and or experience of the intervention from different perspectives: community members, TB suspects and patients referred by storekeepers, storekeepers, community health committees, health workers and policy makers. The trustworthiness of qualitative data collected was assured by the following mechanisms: skilled researchers conducted the data collection and undertook analysis, different sources of data and experiences of community members, storekeepers and health workers were triangulated. The key findings were shared with key informants to check meanings and obtain feedback. All interviews were conducted by BNS, PN and trained research assistants. A total of 55 individual in depth interviews and 20 focus group discussions were conducted.

### Qualitative Analysis

Permission was obtained from the participants to tape record all the FGDs and interviews. Verbatim transcription of the tapes was undertaken and translated from Chichewa to English. Notes from the field were written up and used in analysing the FGDs and IDIs transcripts. In line with the framework approach to qualitative analysis, data were classified into themes and sub-themes [18] using MAXQDA software.

### Ethical Consideration

Ethical approval was granted by the Malawi National Health Science Research Committee and Liverpool School of Tropical Medicine Research Ethics Committee. The informed verbal consent was sought from all individuals who participated in the study and for recording to take place as the literacy level is low in Malawi. Confidentiality was observed by using codes for qualitative transcripts.

## Results

### Chronic Cough Surveys Results: Description of Study Participants

In the intervention sub-districts, 2400 households were screened in 2003 and then again in 2006. In the control sub-district, 1200 households were screened in 2003 and then again in 2006. A total of 540 people (356 in intervention and 184 in comparison areas) suffering from chronic cough were identified and interviewed in 2003. The characteristics of the surveyed community members suffering from chronic cough are shown in Table 3. Notably more women were identified in both years in intervention and comparisons areas.

**Table 3:** Characteristics of Participants in Chronic Cough Household Survey.

	Intervention Group 2003	Intervention Group 2006	Comparison Group 2003	Comparison Group 2006
Sample size	356	347	184	108
Mean age	29.9	28.3	31.0	33.2
Male	69 (19.4% CI 14–26.1%)	66 (19.0% CI 13.6–25.8%)	45 (24.4% CI 16.4–34.7%)	19 (17.6% CI 9.0–30.8%)
Female	287 (80.6% CI 73.9–86.0%)	291 (83.9% CI 77.3–88.8%)	139 (75.5% CI 65.3–83.6%)	89 (82.4% CI 69.2–91%)
Poverty rate	320 (89.9% CI 84.2–93.7%)	310 (89.35% CI 83.5–93.3%)	180 (97.8% CI 91.6–99.6%)	98 (90.7% CI 78.9–96.5%)

#### Chronic Cough Survey: Care seeking pathway and sputum test results

Table 4 summarises key findings on the care seeking pathway among study participants in the household surveys. In the intervention areas 56% (CI:50.2–61.9) sought care from grocery shops in 2003 and 75.5% (68.3–81.6) in 2006. In the comparison area, 60% (CI: 54.3–68.4%) in 2003 and 66.7% (CI:52.4–78.5) in 2006 sought care from the grocery shops as first provider.

**Table 4:** Chronic Cough Survey: Care seeking, Delay, Referral by storekeepers.

	Intervention Group 2003	Intervention Group 2006	Comparison Group 2003	Comparison Group 2006
Sample size	356	347	184	108
Proportion who visited grocery shops first for medication	56% (CI:50.2–61.9)	75.5% (CI:68.3–81.6)	60% (CI:54.3–68.4%)	66.7% (CI: 52.4–78.5)
Number (% and 95% Confidence Intervals) visiting grocery shops given advice and referred	1 (0.3%) CI:0–3.11%	147 (42.4%) CI: 37.1–47.8%	1 (0.54%) CI: 0–5.9%	3 (2.8%) CI:0.3–12.5%
Number (% and 95% Confidence Intervals) seeking care from public health facility at one point of pathway	65 (18.3%) CI:14–22.2%	317 (91.4%) CI: 88.7–94.6%	44 (23.9%) CI: 17.5–30.4%	55 (50.9%) CI:37.1–64.6%
Mean delay (weeks) (Standard Deviation (SD))	8.43 (SD 10.5)	2.14 (SD 5.9)	9.33 (SD 14.5)	8.79 (SD 15.1)
Number (% and 95% Confidence Intervals) of patients who submitted sputum during first visit to a health facility	1/65 (1.5% CI:0.04–8.3%)	37/317 (11.6% CI: 7.3–18%)	0/44 0.0%	3/55 (5.5% CI:0.3–12.5%)
Number (% and 95% Confidence Intervals) of chronic cough cases who started TB treatment	0/356	14/347 (4.0% CI: 1.8–8.5%)	0/184 0.0%	2/108 (1.9% CI:0.09–11.2%)
Generalise Linear Model results- Effect of intervention on delay				
Coefficient	Robust Standard Error	Z	P>|z|	Confidence Interval
1.42	0.58	2.46	0.01	0.29 to 2.55
*After adjusting for age, activity for livelihood*				
1.18	0.65	1.81	0.07	−0.096 to 2.46

At baseline, the proportion of patients either referred or advised by storekeepers in the survey sample in both intervention and comparison group were similar; less than 1%. In 2006 the proportion of study participants suffering chronic cough and seeking medication from grocery shops who were given advice or referral letters was 56 percent (CI: 47–64.7%) in intervention areas compared to 2.8% (CI: 0.3–12.5%) in the comparison area.

Before the intervention in 2003, the mean patient delays were similar in the intervention and comparison areas (8.4 weeks (standard deviation (SD) 10.5) in intervention and 9.3 weeks (SD 14.5 in comparison). Following the intervention in 2006, the mean patient delay was lower in the intervention than comparison areas (2.14 weeks (SD 5.8) vs 8.8 weeks (SD 15.1). The crude effect of the intervention in reducing delay was 41% (p = 0.01). However, after adjusting for sex, age, type of livelihood activity and education level of the patients, the effect was 18% which was not significant (p = 0.07). The proportion of chronic cough cases who submitted sputum during the first visit to the health facility was significantly higher in intervention areas at 11.6% compared to 2.7% (p = 0.003) in comparison sites in 2006 following the intervention. No observation was made in 2003.

### Health Centre Routine Data: TB suspects and sputum results

All health facilities in Lilongwe were audited in order to identify all cases from the study areas. A total of 4225 TB suspects (2.1% from intervention areas and 2.6% from control area) and 1132 (1.6% from intervention and 2.6% from control areas) smear positive TB cases were audited from health centre registers in entire urban Lilongwe in 2003. In 2006, the audit of routine TB registers for all of urban Lilongwe revealed 4625 TB suspects. The smear positive TB cases for urban Lilongwe were 1093 (4.5% from the intervention areas and 3.3% from the comparison area).

During the pre-intervention period there was no difference in the number of cases per 1000 of the population submitting sputum for TB diagnosis between the intervention and comparison areas (3.4 per 1000 and 3.1 per 1000 respectively [chi-square 0.37, p = 0.54] see Table 5). In 2006 the number of cases per 1000 was significantly higher in the intervention areas (6.9 per 1000) than in the comparison areas (2.9 per 1000, p<0.001). In 2003 there was no difference in the incidence of smear positive TB between intervention and comparison sites (0.9 and 0.6 per 1000 respectively). In 2006 the incidence of smear positive TB in the intervention sites (1.2 per 1000) was significantly higher than in the comparison area (0.6 per 1000, p<0.01) even after adjusting for sex and age.

**Table 5:** Suspected TB patients registered and sputum results.

	Intervention Group 2003	Comparison Group 2003	P-value	Intervention Group 2006	Comparison Group 2006	P-value
Population	26,805	34,692		40,500^1^	60,617^2^	
Number of TB suspects tested	88 (3.3/1000 CI:2.8–4.2)	108 (3.1/1000 CI:2.5–3.8)	*X* *^2^* = 0.37 p = 0.54	281 (6.9/1000 CI:6.1–7.8)	179 (3.0/1000 CI:2.5–3.4)	*X* *^2^* = 86, p = 0.00
Mean age (Standard Deviation)	35.2 (SD 10.2)	32.6 (SD 11)		35 (SD 11.5)	34.9 (SD 11.7)	
Patients with smear positive test	18 (0.7/1000 CI: 0.4–1.4)	30 (0.9/1000 CI: 0.6 1.3)	*X* *^2^* = 0.9,p = 0.3	49 (1.2/1000 CI: 0.9–1.6)	36 (0.6/1000 CI: 0.4–0.8)	*X* *^2^* = 10.9, p = 0.00
Mean age (Standard Deviation)	32.6 (SD 11.0)	32.9 (SD 8.3)		36.3 (SD 11.9)	32.7 (SD 11.4)	

1 2007 population projection.

2 2007 population projection.

### Qualitative Results: Perceptions on Role of Storekeepers

The storekeepers were regarded by community members as informal health providers used by communities for cough and other illnesses in intervention and comparison areas. Community members mentioned that the poorest and poor use storekeepers more as the first source of care for most illnesses compared to the non-poor. Participants claimed that non-poor can afford medical insurance which reduces costs of care compared to poor community members. An illustrative quote to highlight this theme is as follows:

*“Since we have different financial capacity, the poor who cannot afford transport visit the storekeepers more times because they cannot go to the hospital” (interventionwomenfocus group discussion 4)*

Differences in perceptions on patterns of use of storekeepers were noted according to the gender of the participant in all communities. Men mainly talked about storekeepers in relation to buying drugs for their own illnesses and women for both the children and themselves.

After the intervention there were changes in perception of the storekeepers' approaches in delivering health services to community members in the intervention sites. Community members positively acknowledged the referral and advice provided by storekeepers in intervention areas.

*Unlike in the past when we visit some storekeepers these days they give us a referral letter to go to the hospital and we then get treated at the hospital. (Intervention women focus group group2006)*

#### Factors Contributing to Acceptance of Referral Role of Storekeepers

The policy makers, health workers and storekeepers shared the view that the referral system improved the access of community members to TB diagnostic centres. The health workers working in the TB registry noted that the referral letter triggered initiation of the TB diagnosis process when presented to health workers. This also led to early initiation of treatment especially for smear positive cases. The project was described as reaching out to people who faced challenges in accessing health care. One of the health workers observed:

*“…the intervention has shown us that when we go to communities and work with informal providers we can improve on our key TB services i.e. case finding and short-course treatment”. (indepthinterviewhealthworker 24)*

The referral process was very popular due to fast response from the health facility. The referral process was referred to as having a positive impact on doctors who are “happy because they receive patients before reaching a critical condition” (Storekeeper Ngwenya12006).

#### Improved Access to TB Diagnosis and Treatment

Patients diagnosed with TB in the intervention areas indicated that the referral system resulted in early access to services. All participants mentioned that during the first visit to the trained storekeepers they were given referral a letter to go to the health facility for diagnosis.

*“He did not sell me any drug; he insisted that I should go to the hospital for proper investigation and treatment” (Femaleinterventionin-depth interview2006)*

Testimonies by the patients in the interviews showed that health workers gave them priority and the health facility was able to respond to them quickly. For example a typical quote:

*“When you are given a referral letter you don't stand on a line, you just go straight to the health worker like one of the staff” (In-depth interview female respondent Ngwenya 6)*

Patients also acknowledged that the referral letters were helpful in accessing TB diagnostics:

*“Initially I was self treating myself by taking panado(paracetamol)* [*1*] *and when I went to store (Chilungamo) where I was given a referral letter and went to the hospital and explained to them my problem. They asked me to submit sputum. At first the results were negative. Then I went back and I was referred for an X-Ray and found that I have TB”. (Female in-depth interview Ngwenya 3)*

Although the response of health workers improved towards people with chronic cough, those who submitted sputum with negative results and were still coughing were worried about their situation. Some of them also acknowledged that the health workers gave them drugs to treat the cough.

*“When I went to the health facility I was given bottles for sputum submissions. When I went to get the results I was not diagnosed with TB. I was only given drugs and was told might be asthma” (Male respondent Ngwenya in-depth interview 5)*

#### Motivation Factors for Storekeepers at Community level

The storekeepers felt that their advisory and referral skills made them popular in the community. Some reported that they were motivated especially when referred customers were appropriately diagnosed and treated. The storekeepers indicated that the training and review meetings were good motivating factors. In addition the business management training was perceived to have improved their recording and marketing skills.

#### Challenges Related to the Intervention

The community members and leaders in focus group discussions and in depth interviews highlighted that some of the poorest referred to health facilities faced difficulties going to the hospital because of lack of transport, distance and money. They mentioned that in circumstances of extreme poverty opportunity costs to visit a health facility were high. People had to make difficult choices between losing a day's work or going to the health facility. Typical views from participants in the focus group discussions include:

*“Sometimes it happens that a person has been given referral letter and is coughing but you will find that he is just walking around you see until the cough reaches to an advanced stage. This is mainly due to extreme poverty which hinders people to raise transport money and avoid spending time waiting at the health facility compared to working to earn money for the family. Poverty is also a contributing factor, a person maybe suffering but since he doesn't have anything to support himself at home, because of that he just decides to go somewhere to look for food and there it means days are elapsing. Due to that you will find that the disease has reached to an advanced stage”. (Kauma Men focus group 1)*

It was also noted by community members that the first contact at the health facility when given a referral is the outpatient department which sometimes has insufficient drugs. This disappointed both the community members and storekeepers, for example:

*“It happens you have spent transport money in going to the hospital and you will find that they are giving you Aspirin instead of using that money to buy other painkillers to relieve that cough. So I would like to ask you on some other time when you come here to test cough, you should also be giving us treatment because we sometimes spend money at the hospital but we are not given treatment.” interventionwomenwomenfocusgroupdiscussion20063)*

Another challenge was turnover of staff in the Out Patient Department (OPD) as sometimes referred patients were turned away by new health workers. However the staff responsible for the chronic cough registry and laboratory did not change throughout the lifespan of the project. This provided a strong base for the institutional memory of the intervention model and systems and they acted as mentors for their colleagues from the City Assembly and OPD.

## Discussion

The key result of this study is that smear positive TB cases detected from the intervention areas increased. Both quantitative and qualitative results showed that storekeepers are a key provider of medicines for patients seeking diagnosis for chronic cough. Triangulation of results from different sources of data such as key informants and community members revealed general agreement that the referral system was effective in reducing delay and triggering health workers to request patients to submit sputum for TB diagnosis. As documented by studies in China and Vietnam, delay occurs between consultation and TB diagnosis units, and this study illustrates one approach to address barriers to achieving a diagnosis [4]–[5]. The quantitative aspect of the evaluation showed an increase in the number of TB cases and a reduction in delay. The participatory training led to empowerment of storekeepers in advising and referring their clients with chronic cough. Other studies have also shown that provider and health system delays are longer for patients who are not diagnosed or referred at their first visit to any health provider [18]. The referral system with storekeepers intervened by triggering health workers to think about TB diagnosis during the first visit to a formal health facility. The Malawi National TB Control Programme adapted the approach for scale-up by developing a manual and guidelines for district health offices. The study also adds to knowledge about options for partnerships in increasing entry points for early access to diagnosis and treatment. Within the framework of private public partnership promoted by World Health Organisation, integration of storekeepers who are prominent in settings like Malawi can increase case detection and contribute to meeting of Global TB and national TB Control goals.

The acceptability of the intervention among providers and community members was good. No major variations in perceptions and views about its impact were noted from the qualitative study. The key underlying factors of acceptance were impact on health workers' decision to request sputum submission and sometimes queuing was not required as patients were directly sent to TB registries. Policy makers and health workers accepted the intervention because it was perceived to have a positive impact on case detection which is associated with systems performance. An intervention can only be acceptable and sustainable if it addresses the interest of all partners from their perspectives.

When informal providers operate within the scope of their livelihood, there are limited barriers to sustainability. The storekeepers were not taken away from their day to day livelihood activities, instead the referral system built on this as a strength for sustainability. In this study the major motivation factors for storekeepers' participation were social recognition, training and support from health workers. The storekeepers' referral role enhanced their social status in the community. The linkage of storekeepers' role to their livelihood was an important factor for sustainability of intervention. In contexts like Malawi where human resources for health are scarce, WHO and other partners are promoting task shifting as a new concept to ensure accessibility of life-saving interventions such as access to counselling and testing and antiretroviral therapy for HIV. However working with volunteers with no or limited livelihoods adds financial burdens on their families. Storekeepers are self-employed and in this case their health role was linked to their livelihood strategy. There have been proposals of financial reward to community workers in the HIV and AIDS sector to avoid impacting negatively on the livelihoods of volunteers but sustainability of this approach is not clear in the context of limited resources [19]–[21].

The process of consultation with leaders, community-based informal providers, health workers and policy makers provided additional insights into roles that are acceptable and based on existing responsibilities of different stakeholder within their communities and health facilities. Although the process was lengthy it was necessary in building the rapport between the health workers, community leaders and community informal providers.

Most evaluations of health system interventions do not consider documenting processes and social-contextual factors which are critical in shaping project success. Qualitative research is critical in unpacking social contexts and understanding phenomena in its natural setting [22]. In the context of our study it provided key findings related to drivers of acceptability of the intervention at community, health facility and policy levels.

## Conclusions

The study has shown that the referral system increase smear positive TB case detection in the intervention areas. The capacity building activities equipped storekeepers with communications skills enabling them to identify and refer their customers who were suffering from chronic cough. The qualitative assessment documented factors that underpinned the change in skills and practice of storekeepers. In addition the assessment also mapped out factors that motivated participation of the storekeepers which were recognition of their time during training, acknowledgment by their communities on their contribution and the certification process. In contexts where they are regularly accessed, storekeepers should be considered as key players in community and public-private partnership models to enhance case finding amongst poor and vulnerable groups. Future research should focus on integrating other diseases and collection of diagnostic specimens by storekeepers or similar providers.
